# Клинические рекомендации «Врожденный гипотиреоз»

**DOI:** 10.14341/probl12880

**Published:** 2022-04-30

**Authors:** В. А. Петеркова, О. Б. Безлепкина, Т. Ю. Ширяева, Т. А. Вадина, Е. В. Нагаева, О. А. Чикулаева, Е. В. Шредер, М. Б. Конюхова, Н. А. Макрецкая, Е. А. Шестопалова, В. Б. Митькина

**Affiliations:** Национальный медицинский центр эндокринологии; Национальный медицинский центр эндокринологии; Национальный медицинский центр эндокринологии; Национальный медицинский центр эндокринологии; Национальный медицинский центр эндокринологии; Национальный медицинский центр эндокринологии; Национальный медицинский центр эндокринологии; Московский центр неонатального скрининга Морозовской детской городской клинической больницы; Национальный медицинский центр эндокринологии; Медико-генетический научный центр имени академика Н.П. Бочкова; Московский центр неонатального скрининга Морозовской детской городской клинической больницы

**Keywords:** клинически рекомендации, врожденный гипотиреоз, дети

## Abstract

Врожденный гипотиреоз является актуальной проблемой детской эндокринологии, но своевременная диагностика и лечение позволяют предотвратить развитие тяжелых случаев заболевания. Разработанные клинические рекомендации являются рабочим инструментом практикующего врача, их целевая аудитория — это детские эндокринологи и педиатры. В них кратко и логично изложены основные положения об определении заболевания, эпидемиологии, классификации, методах диагностики состояния и лечения, базирующихся на принципах доказательной медицины.

## Abstract (en)

Congenital hypothyroidism is an important issue of pediatric endocrinology at which timely diagnosis and treatment can prevent the development of severe cases of the disease. The developed clinical guidelines are a working tool for a practicing physician. The target audience is pediatric endocrinologists and pediatricians. They briefly and logically set out the main definition of the disease, epidemiology, classification, methods of diagnosis and treatment, based on the principles of ­evidence-based medicine.

## СПИСОК СОКРАЩЕНИЙ

ВГ — врожденный гипотиреозГГС — гипоталамо-гипофизарная системаГП — гипопитуитаризмЛГ — лютеинизирующий гормонМРТ — магнитно-резонансная томографияППР — преждевременное половое развитиеТГ — тиреоглобулинТРГ — тиреотропин-рилизинг-гормонТТГ — тиреотропный гормонТАБ — тонкоигольная аспирационная биопсияУЗИ — ультразвуковое исследованиеФСГ — фолликулостимулирующий гормонЧСС — частота сердечных сокращенийЩЖ — щитовидная железаЭКГ — электрокардиографияЭхоКГ — эхокардиографияSD — стандартное отклонениеSDS — коэффициент стандартного отклоненияT3 — трийодтиронинT4 — тироксинЦНС — центральная нервная системаIQ — intelligence quotient (c англ. коэффициент интеллекта)WISC — Wechsler Intelligence Scale for Children (тест Векслера)

## ТЕРМИНЫ И ОПРЕДЕЛЕНИЯ

Гипотироксинемия — недостаточность тиреоидных гормонов.

Неонатальный скрининг — массовое обследование всех новорожденных детей на гипотиреоз с определением ТТГ в капиллярной крови, позволяющее выявить большинство случаев заболевания на доклиническом этапе и своевременно назначать заместительную терапию.

Дисгенезия ЩЖ — структурное нарушение щитовидной железы, связанное с дефектом эмбрионального развития тиреоидной ткани, проявляющееся аплазией, гемиагенезией, гипоплазией или эктопией (дистопией).

Дисгормоногенез — нарушение выработки или транспорта тиреоидных гормонов, возникающее вследствие ферментативных дефектов (органификации йода, синтеза тиреоглобулина, тиреопероксидазы и т. д.).

Первичный гипотиреоз — клинический синдром, развивающийся вследствие недостаточной продукции тиреоидных гормонов по причине первичной патологии в самой ЩЖ.

Вторичный гипотиреоз — клинический синдром, развивающийся вследствие недостаточной продукции ТТГ при отсутствии первичной патологии самой ЩЖ, приводящей к снижению ее функции.

Транзиторный гипотиреоз — состояние временной гипотироксинемии, сопровождающееся повышением ТТГ.

## 1. КРАТКАЯ ИНФОРМАЦИЯ ПО ЗАБОЛЕВАНИЮ ИЛИ СОСТОЯНИЮ

## 1.1. Определение заболевания или состояния

Врожденный гипотиреоз (ВГ) — одно из наиболее часто встречающихся врожденных заболеваний ЩЖ у детей, в основе которого лежит полная или частичная недостаточность тиреоидных гормонов, приводящая к задержке развития всех органов и систем организма при отсутствии своевременно начатого лечения [1].

## 1.2. Этиология и патогенез заболевания или состояния (группы заболеваний или состояний)

ВГ — гетерогенная по этиологии группа заболеваний, обусловленных чаще всего морфофункциональной незрелостью ЩЖ, реже — гипоталамо-гипофизарной системы (ГГС).

Гипотироксинемия приводит к развитию метаболических нарушений, снижению скорости окислительных процессов и активности ферментных систем, повышению трансмембранной клеточной проницаемости и накоплению в тканях недоокисленных продуктов обмена. Дефицит тиреоидных гормонов грубо нарушает процессы роста, дифференцировки всех тканей и систем организма.

Больше других от недостатка тиреоидных гормонов у ребенка страдает центральная нервная система. Низкий уровень тиреоидных гормонов, особенно в первые месяцы жизни, приводит к задержке процессов миелинизации нервных волокон, снижению накопления липидов и гликопротеидов в нервной ткани, что в итоге вызывает морфофункциональные нарушения в мембранах нейронов проводящих путей мозга.

Необратимость повреждений ЦНС при врожденном гипотиреозе в условиях отсутствия лечения связана с особенностями роста и созревания головного мозга новорожденного.

В период максимального роста и активного нейрогенеза, который приходится на первые 6 мес жизни ребенка, мозг оказывается особенно чувствителен к неблагоприятным воздействиям, в том числе и к недостатку тироксина. Поэтому тиреоидная недостаточность в критическом периоде наиболее быстрого развития ЦНС задерживает ее созревание, приводя к необратимой умственной отсталости [2–6].

## 1.3. Эпидемиология заболевания или состояния (группы заболеваний или состояний)

Частота врожденного гипотиреоза колеблется от 1:3000–4000 новорожденных в Европе и Северной Америке до 1:6000–7000 новорожденных в Японии. У лиц негроидной расы заболевание встречается достаточно редко (примерно 1:30 000), а у латиноамериканцев, напротив, часто (1:2000). У девочек заболевание встречается в 2–2,5 раза чаще, чем у мальчиков. Распространенность ВГ в Российской Федерации, по результатам неонатального скрининга, составляет 1 случай на 3600 новорожденных (1997–2015) [2][7].

## 1.4. Особенности кодирования заболевания или состояния (группы заболеваний или состояний) по Международной статистической классификации болезней и проблем, связанных со здоровьем

E03.0 Врожденный гипотиреоз с диффузным зобом. Зоб (нетоксический) врожденный паренхиматозный.

Е03.1 Врожденный гипотиреоз без зоба. Аплазия щитовидной железы (с микседемой). Врожденная атрофия щитовидной железы.

Е07.1 Дисгормональный зоб. Семейный дисгормональный зоб. Синдром Пендреда.

Е07.8 Другие уточненные болезни щитовидной железы. Дефект тироксинсвязывающего глобулина. Кровоизлияние в щитовидную железу. Инфаркт щитовидной железы. Синдром нарушения эутиреоза.

## 1.5. Классификация заболевания или состояния (группы заболеваний или состояний)

По уровню поражения (наиболее распространенная на сегодняшний день).

Первичный гипотиреоз.

Дисгенезия щитовидной железы (нарушение строения и закладки):

Дисгормоногенез (нарушение синтеза тиреоидных гормонов):

Центральный гипотиреоз (вторичный, третичный):

Периферическая резистентность к тиреоидным гормонам (мутации генов TRHA и TRHB).

Транзиторный гипотиреоз.

По степени тяжести.

Латентный (субклинический) — повышенный уровень ТТГ при нормальном уровне свободного тироксина (T4).

Манифестный — гиперсекреция ТТГ при сниженном уровне свободного T4, наличие клинических проявлений.

Тяжелого течения (осложненный), при котором может быть кретинизм, сердечная недостаточность, выпот в серозные полости, вторичная аденома гипофиза.

По степени компенсации:

Осложненный гипотиреоз (как правило, не распознанные вовремя, запущенные случаи заболевания) без своевременно назначенной и правильно подобранной заместительной медикаментозной терапии может привести к развитию гипотиреоидной (микседематозной) комы.

В подавляющем большинстве случаев (85–90%) имеет место первичный ВГ. Среди первичного гипотиреоза 85% случаев являются спорадическими, большинство из них обусловлено дисгенезией (эмбриопатией) щитовидной железы (ЩЖ). По данным различных авторов, агенезия ЩЖ встречается в 22–42% случаев, в 35–42% случаев ткань железы эктопирована, в 24–36% имеет место гипоплазия ЩЖ [8–12].

Гораздо реже (5–10%) встречаются вторичный или третичный ВГ, проявляющиеся изолированным дефицитом ТТГ или гипопитуитаризмом (ГП) [9–13].

Особой формой ВГ является транзиторный гипотиреоз новорожденных. Эта форма заболевания чаще всего наблюдается в регионах, эндемичных по недостатку йода. Транзиторный гипотиреоз может возникнуть и в результате незрелости системы органификации йода, особенно у недоношенных, незрелых новорожденных. К развитию транзиторного гипотиреоза у новорожденного может приводить прием матерью во время беременности тиреостатических и других препаратов, нарушающих синтез тиреоидных гормонов ЩЖ плода. Описана трансплацентарная передача материнских блокирующих антител к рецептору ТТГ [1].

В связи с развитием методов молекулярно-генетического анализа взгляды на этиологию врожденного гипотиреоза в последние годы во многом изменились. На сегодняшний день идентифицирован ряд генов, мутации которых приводят к нарушениям закладки, миграции, дифференцировки ЩЖ, дефектам синтеза тиреоидных гормонов, нарушениям гипоталамо-гипофизарной оси. Отсутствие специфических симптомов, характерных для определенного генетического дефекта, не позволяет проводить изолированную диагностику одного гена для идентификации мутации. Наиболее широко изучены варианты дисгенезии ЩЖ, однако показано, что нарушение закладки этого жизненно важного органа ассоциировано с мутациями генов только в 2% случаев, в остальных случаях причина остается неизвестной. В структуре наследственных форм заболевания преобладающими причинами развития ВГ являются дефекты генов дисгормоногенеза, о чем свидетельствуют результаты молекулярно-генетического анализа [10–17].

## 1.6. Клиническая картина заболевания или состояния (группы заболеваний или состояний)

Клинические проявления гипотиреоза.

Клинические проявления и течение гипотиреоза существенно различаются у лиц разного возраста [1][14–18]. В детском возрасте они зависят от периода манифестации заболевания, длительности синдрома гипотиреоза и сроков начала заместительной терапии. На 1-м месяце жизни ребенка, когда ранняя диагностика крайне важна, типичная клиническая картина ВГ наблюдается всего в 10–15% случаев.

ВГ у новорожденных проявляется следующими симптомами:

У детей более старшего возраста (после 5–6 мес) клинические проявления гипотиреоза схожи с проявлениями у взрослых. Помимо этого, при отсутствии лечения у детей с ВГ на первый план выступает нарастающая задержка психомоторного, физического, а затем и полового развития.

Отстает развитие моторики:

Кожные покровы:

Характерен комплекс респираторных симптомов:

Выражены:

Характерны:

Характерны:

Характерны:

Транзиторный гипотиреоз новорожденных — состояние временной (преходящей) гипотироксинемии, сопровождающееся повышением уровня ТТГ в крови. Транзиторное повышение уровня ТТГ в большинстве случаев связано с функциональной незрелостью гипоталамо-гипофизарной системы в постнатальном периоде.

Данное состояние чаще всего встречается в следующих случаях:

На этапе первичного скрининга практически невозможно различить врожденный и транзиторный гипотиреоз. Разграничение этих состояний необходимо проводить на II этапе скрининга, то есть в поликлинических условиях, при повторном определении концентраций ТТГ и свободного T4 в сыворотке на фоне отмены заместительной терапии.

Предикторы транзиторного гипотиреоза:

Вторичный гипотиреоз чаще всего является следствием гипопитуитаризма (ГП), поэтому наличие других типичных симптомов ГП (пороки развития головного мозга и черепа, гипогликемии, микропения, крипторхизм у мальчиков) позволяет заподозрить правильный диагноз. Вторичный гипотиреоз, обусловленный нарушением функции аденогипофиза или гипоталамуса (мутации генов Pit-1, PROP-1), сопровождается дефицитом не только ТТГ, но и других тропных гормонов. Врожденный изолированный дефицит ТТГ — крайне редкое аутосомно-рецессивное заболевание, причиной которого являются мутации гена TSH α- и β-субъединиц [15–17][19–23].

По сравнению с первичным, вторичный гипотиреоз характеризуется более стертой и мягкой клинической картиной. При вторичном гипотиреозе концентрации общего и свободного T4 снижены, а уровень ТТГ может быть умеренно повышенным, нормальным или сниженным. Транзиторный вторичный гипотиреоз чаще выявляют у недоношенных и маловесных новорожденных. Он может быть обусловлен незрелостью ГГС или ГП. Отличить истинный вторичный гипотиреоз от транзиторного вторичного гипотиреоза очень сложно. Снижение уровней T4 и T3 у недоношенных новорожденных отражает их адаптацию к стрессу и не является показанием для заместительной терапии тиреоидными гормонами. К 1–2-му месяцу жизни уровни T4 и T3 в сыворотке постепенно увеличиваются и достигают нормальных значений, характерных для доношенных детей того же возраста. Истинные нарушения функции ЩЖ у таких детей можно выявить после нормализации их веса и развития [18][20][24][25].

## 2. ДИАГНОСТИКА ЗАБОЛЕВАНИЯ ИЛИ СОСТОЯНИЯ

Критерии установления диагноза.

Основная цель скрининга на ВГ — максимально раннее выявление всех новорожденных с повышенным уровнем ТТГ в крови. Дети с аномально высоким уровнем ТТГ требуют в дальнейшем углубленного обследования для правильной диагностики заболевания [6][14][20][21–32].

Рекомендуется диагностировать ВГ у новорожденных согласно результатам неонатального скрининга на ВГ и/или исследования уровня ТТГ в крови, уровня Т4св.) сыворотки крови [1][2][14][21–27] (УУР — С; УДД — 3).

Рекомендуется исследование уровня ТТГ в крови у новорожденного не позднее 5 сут жизни (оптимальный срок — полные 3-и сутки) в пятне цельной крови [1][2][14][21–27] (УУР — С; УДД — 4).

Рекомендовано обследование и дальнейшее наблюдение детей в три этапа [1][2][14][21–27].

I этап — родильный дом, стационар, детская поликлиника.

У всех доношенных новорожденных анализ крови на скрининг (капиллярная кровь из пятки) берут не позднее 5-х суток жизни (оптимально по прошествии полных 3-х суток с момента рождения), у недоношенных детей — на 7 и 14-й день жизни; капли (в количестве 6–8 капель) наносятся на специальную пористую фильтровальную бумагу.

Все образцы крови отсылают в специализированную медико-генетическую лабораторию.

II этап — медико-генетическая лаборатория.

В лаборатории проводят определение концентрации ТТГ в сухих пятнах крови.

Для диагностики ВГ применяется массовое определение ТТГ в капиллярной крови (например, АвтоДелфия Нео-тиреотропный гормон, метод флюориметрического анализа). Пороговые значения ТТГ определяются наборами применяемых тест систем в каждой лаборатории.

1. ТТГ капиллярной крови менее 9 мЕд/л у доношенного ребенка в возрасте 4–14 дней считается нормой.

2. ТТГ капиллярной крови выше 9 мЕд/л у доношенного ребенка в возрасте 4–14 дней требует повторного определения ТТГ из того же образца крови, при получении аналогичного результата проводят срочное уведомление ЛПУ для повторного забора крови (ретест) и доставки образца капиллярной крови в лабораторию неонатального скрининга для определения уровня ТТГ.

А. ТТГ капиллярной крови от 9,0 до 40,0 мЕд/л: в лаборатории повторно определяют ТТГ из того же образца крови, при получении аналогичного результата проводят срочное уведомление поликлиники и забор венозной крови для определения ТТГ и Т4св. в сыворотке или ретестирование (повторный забор капиллярной крови).

Б. ТТГ капиллярной крови более 40,0 мЕд/л: в лаборатории повторно определяют ТТГ из того же образца крови, при получении аналогичного результата — проводят срочное уведомление поликлиники и забор венозной крови для определения ТТГ и свободного Т4 в сыворотке. Не дожидаясь результатов, назначается заместительная терапия тиреоидными препаратами, при невозможности получения результатов в день забора крови. Если полученные результаты окажутся в пределах нормальных значений, терапия будет отменена [1][19].

Интерпретация результатов ретестирования в капиллярной крови:

Интерпретация результатов исследования венозной крови (уточняющая диагностика):

III этап — детская поликлиника.

На этом этапе за детьми с ВГ, выявленным по результатам неонатального скрининга, ведется динамическое наблюдение врачами-детскими эндокринологами [1][19] (УУР — С; УДД — 4).

## 2.1. Жалобы и анамнез

## 2.1.1. Период новорожденности

Жалобы:

В анамнезе — переношенная беременность.

## 2.1.2. 1-й год жизни

Жалобы:

## 2.1.3. Дошкольный и младший школьный возраст

Жалобы:

## 2.1.4. Старший возраст

Жалобы:

Рекомендовано: сбор подробного анамнеза и жалоб у пациента для правильной постановки диагноза и назначения лечения. Необходимо обращать внимание на клинические симптомы гипотиреоза [1][19] (УУР — С; УДД — 5).

## 2.2. Физикальное обследование

ВГ у новорожденных проявляется следующими симптомами:

Рекомендуется для диагностики ВГ у новорожденных педиатрам, неонатологам и эндокринологам использовать шкалу Апгар для детей в ВГ, помогающую заподозрить заболевание в ранние сроки. При сумме баллов более 5 следует заподозрить ВГ [1] (УУР — С; УДД — 5).

## 2.3. Лабораторные диагностические исследования

Гормональные исследования.

Пациентам с ВГ рекомендовано:

Дополнительные гормональные исследования.

Пациентам с ВГ рекомендовано по показаниям:

Ультразвуковое исследование ЩЖ.

УЗИ ЩЖ рекомендовано пациентам с ВГ:

Сцинтиграфия ЩЖ (с натрия пертехнетатом [ 99mTc]).

Сцинтиграфия ЩЖ рекомендована пациентам с ВГ при аплазии или эктопии по результатам УЗИ ЩЖ:

Можно проводить всем детям с ВГ, независимо от возраста, в том числе новорожденным.

Если при проведении сцинтиграфии ЩЖ не визуализируется, диагноз не вызывает сомнений. Этот метод исследования (в отличие от УЗИ) позволяет выявить дистопически расположенную ткань ЩЖ (УУР — С; УДД-4).

Выполнение данной процедуры не должно мешать своевременном старту лечения левотироксином натрия. Оно проводится в короткие сроки до начала или в течение 7 дней после инициации заместительной гормональной терапии, либо на фоне отмены терапии в течение 2–3 нед. Применение натрия пертехнетата [ 99mTc] обосновано свойством клеток ЩЖ накапливать данный радиофармацевтический препарат (наблюдаемый максимум накопления с 10-й по 30-ю минуту после введения), подобно йоду (в синтезе тиреоидных гормонов натрия пертехнетат [ 99mTc] не участвует, так как не подвергается органификации). Технеций обладает коротким периодом полураспада (~6 ч) и, соответственно, достаточно быстро полностью выводится из организма. В настоящее время накоплен длительный опыт (с 1960-х гг.) использования натрия пертехнетата [ 99mTc] в педиатрической практике при многих нозологиях и доказана его безопасность. По сравнению с натрия йодогиппуратом, 123-I, натрия пертехнетат [ 99mTc] применяется значительно чаще, его использование оправдано в первую очередь меньшей лучевой нагрузкой на организм, а также более низкой ценой и доступностью. Установлено, что рудиментарная ткань ЩЖ при ее дистопии способна достаточно длительно продуцировать тиреоидные гормоны, ее функциональная активность значительно снижается после десятилетнего возраста. В этих случаях может быть диагностирован ВГ с поздними проявлениями (поздняя форма ВГ). Существуют различные варианты дистопии ЩЖ: в корень языка или по ходу тиреоглоссального протока, при этом может наблюдаться самая различная степень тяжести ВГ [1][2][19][33][34].

Молекулярно-генетическое исследование.

Молекулярно-генетическое исследование рекомендовано пациентам с ВГ после медико-генетического консультирования в семейных случаях заболевания или при сочетании с другой органной патологией:

Показана высокая значимость молекулярно-генетического исследования для установки точного этиологического диагноза, результаты которого могут быть использованы при проведении пренатальной диагностики в случае подтверждения биаллельных мутаций или при доказанном доминантном наследовании заболевания (PAX 8, NKX 2-1).

## 3. ЛЕЧЕНИЕ, ВКЛЮЧАЯ МЕДИКАМЕНТОЗНУЮ И НЕМЕДИКАМЕНТОЗНУЮ ТЕРАПИИ, ДИЕТОТЕРАПИЮ, ОБЕЗБОЛИВАНИЕ, МЕДИЦИНСКИЕ ПОКАЗАНИЯ И ПРОТИВОПОКАЗАНИЯ К ПРИМЕНЕНИЮ МЕТОДОВ ЛЕЧЕНИЯ

## 3.1. Консервативное лечение

Рекомендовано пациентам с ВГ:

Критерии адекватности лечения ВГ:

**Table:** Таблица 1. Ориентировочные расчетные дозы левотироксина натрия у детей с ВГ [1]Table 1. Approximate calculated doses of levothyroxine sodium in children with CH [1]

Возраст	Мкг/кг/сут	Мкг/сут
Недоношенные новорожденные	8,0-10,0	
0-3 мес	10,0-15,0	15,0-50,0
3-6 мес	8,0-10,0	15,0-50,0
6-12 мес	6,0-8,0	50,0-75,0
1-3 года	4,0-6,0	75,0-100,0
3-10 лет	3,0-4,0	100,0-150,0
10-15 лет	2,0-4,0	100,0-150,0
старше 15 лет	2,0-3,0	100,0-200,0

Лечение транзиторного гипотиреоза.

Пациентам с диагнозом «транзиторный гипотиреоз» рекомендован следующий алгоритм ведения.

При получении показателей ТТГ и Т4св. в пределах референсных значений лечение не возобновляют, контрольные осмотры с определением концентраций ТТГ и Т4св. в сыворотке проводят через 2 нед, 1 и 6 мес после прекращения лечения.

Если диагноз ВГ подтверждается, лечение левотироксином натрия продолжают с постоянным контролем за адекватностью терапии [1][19].

Внимание: если уровень ТТГ на фоне терапии когда-либо повышался вследствие недостаточной дозы левотироксина натрия или нарушения схемы его приема, прерывать лечение для уточнения диагноза не рекомендуется. В этом случае диагноз ВГ не вызывает сомнения [1] (УУР — С ; УДД — 5).

## 3.2. Хирургическое лечение

Оперативное лечение при ВГ рекомендуется:

пациентам, имеющим зоб, при наличии:

пациентам без зоба:

## 4. МЕДИЦИНСКАЯ РЕАБИЛИТАЦИЯ И САНАТОРНОКУРОРТНОЕ ЛЕЧЕНИЕ, МЕДИЦИНСКИЕ ПОКАЗАНИЯ И ПРОТИВОПОКАЗАНИЯ К ПРИМЕНЕНИЮ МЕТОДОВ РЕАБИЛИТАЦИИ, В ТОМ  ЧИСЛЕ ОСНОВАННЫХ НА ИСПОЛЬЗОВАНИИ ПРИРОДНЫХ ЛЕЧЕБНЫХ ФАКТОРОВ

Пациентам с ВГ рекомендовано:

Противопоказаний не определено (УУР — С ; УДД — 5).

## 5. ПРОФИЛАКТИКА И ДИСПАНСЕРНОЕ НАБЛЮДЕНИЕ, МЕДИЦИНСКИЕ ПОКАЗАНИЯ И ПРОТИВОПОКАЗАНИЯ К ПРИМЕНЕНИЮ МЕТОДОВ ПРОФИЛАКТИКИ

Диспансерное наблюдение и прогноз.

Пациентам с ВГ рекомендуется постоянное комплексное углубленное наблюдение у специалистов разного профиля (врача-эндокринолога, врача-невролога, врача-сурдолога, логопеда, медицинского психолога (нейропсихолога); оценка интеллектуального развития с применением теста Векслера (детский вариант); при наличии когнитивных нарушений, психических расстройств, пороков развития — консультация врача-психиатра, врача-кардиолога и др.) [1] (УУР — С; УДД — 5).

Прогноз в отношении нейропсихического развития при ВГ зависит от множества факторов. Исследователи во всех странах сходятся во мнении, что определяющую роль для благоприятного прогноза интеллектуального развития ребенка с ВГ, безусловно, играют сроки начала заместительной терапии левотироксином натрия, хотя ряд авторов указывают, что даже при раннем начале лечения у небольшой части детей те или иные нарушения интеллекта все-таки сохраняются. Крайне важным фактором является адекватность лечения на первом году жизни. Таким образом, за некоторым исключением, все дети с ВГ при раннем и адекватном лечении имеют возможность достичь оптимального интеллектуального развития [1][19].

## 6. ОРГАНИЗАЦИЯ ОКАЗАНИЯ МЕДИЦИНСКОЙ ПОМОЩИ

Рекомендуется плановая госпитализации в медицинскую организацию:

Не рекомендуется госпитализация в стационар при возможности достижения компенсации в амбулаторных условиях [1] (УУР — С; УДД — 5).

Показания к выписке пациента из медицинской организации:

Рекомендуется экстренная госпитализация в медицинскую организацию в случае возникновения:

## 7. ДОПОЛНИТЕЛЬНАЯ ИНФОРМАЦИЯ (В ТОМ ЧИСЛЕ ФАКТОРЫ, ВЛИЯЮЩИЕ НА ИСХОД ЗАБОЛЕВАНИЯ ИЛИ СОСТОЯНИЯ)

Дополнительная информация отсутствует.

## ДОПОЛНИТЕЛЬНАЯ ИНФОРМАЦИЯ

Источники финансирования. Работа выполнена по инициативе авторов без привлечения финансирования.

Конфликт интересов. Авторы декларируют отсутствие явных и потенциальных конфликтов интересов, связанных с содержанием и публикацией настоящей статьи.

Участие авторов. Все авторы одобрили финальную версию статьи перед публикацией, выразили согласие нести ответственность за все аспекты работы, подразумевающую надлежащее изучение и решение вопросов, связанных с точностью или добросовестностью любой части работы.

## ПРИЛОЖЕНИЕ А3.

## СПРАВОЧНЫЕ МАТЕРИАЛЫ, ВКЛЮЧАЯ СООТВЕТСТВИЕ ПОКАЗАНИЙ К ПРИМЕНЕНИЮ И ПРОТИВОПОКАЗАНИЙ, СПОСОБОВ ПРИМЕНЕНИЯ И ДОЗ ЛЕКАРСТВЕННЫХ ПРЕПАРАТОВ, ИНСТРУКЦИИ ПО ПРИМЕНЕНИЮ ЛЕКАРСТВЕННОГО ПРЕПАРАТА

**Table:** Таблица 1. Этиология и распространенность основных форм ВГ [1]Table 1. Etiology and prevalence of the main forms of CH [1]

Причины	Частота (%; на число новорожденных)
Первичный гипотиреоз	
1. Дисгенезия щитовидной железы	80-85 (1:4000)
Агенезия (атиреоз)	22-42
Гипогенезия (гипоплазия)	24-36
Дистопия	35-42
2. Дисгормоногенез	10-15
Дефект рецептора ТТГ	4 (1:30 000-1:50 000)
Дефект транспорта йода	редко
Дефект пероксидазной системы	1:26 000
Дефект синтеза тиреоглобулина	1:40 000
Дефект дейодирования	редко
Центральный гипотиреоз (вторичный, третичный)	
Сочетанный дефицит гипофизарных гормонов	5-10 (1:16 000)
Изолированный дефицит ТТГ	1:75 000-1:100 000
Периферическая резистентность к тиреоидным гормонам	1:100 000
Транзиторный гипотиреоз	Неизвестна

**Table:** Таблица 2. Классификация ВГ [1][46]Table 2. Classification of VG [1][46]

1. Первичный гипотиреоз.А. Дисгенезия щитовидной железы: эктопия, аплазия, гипоплазия, гемиагенезия.Ассоциированы с мутациями генов NKX2-1, FOXE1, PAX-8 — в 2% случаев.Причины неизвестны — в 98% случаев.Б. Дисгормоногенез (нарушение синтеза тиреоидных гормонов).Ассоциирован со следующими генетическими дефектами:дефект натрий-йодного симпортера (мутация гена SLC5A5 (NIS));дефекты пероксидазы:дефекты синтеза перекиси водорода (мутации генов DUOX2, DUOXA2, TPO);дефект пендрина (синдром Пендреда — мутация гена SLC26A4 (PDS));дефект синтеза тиреоглобулина (мутация гена TG);дефект дейодирования (мутации гена IYD).В. Резистентность к ТТГ.Ассоциирована с мутациями генов:дефект гена рецептора ТТГ (TSHR);мутации G-протеина: псевдогипопаратиреоз типа 1a.
2. Центральный гипотиреоз (вторичный гипотиреоз).А. Изолированная недостаточность ТТГ (мутации гена, кодирующего α-субъединицу ТТГ).Б. Недостаточность тиреотропин-рилизинг-гормона (ТРГ): изолированная, синдром повреждения гипофизарной ножки, повреждение гипоталамуса (например, гамартома).В. Резистентность к ТРГ (мутации рецептора ТРГ).Г. Гипотиреоз, вызванный недостаточностью факторов транскрипции, вовлеченных в процессы развития или функционирования гипофиза (мутации генов HESX1, LHX3, LHX4, Pit1, PROP1).
3. Периферический гипотиреоз.А. Резистентность к тиреоидным гормонам (мутации генов THRA и TRHB).Б. Нарушение транспорта тиреоидных гормонов (синдром Allan–Herdon–Dudley — мутация гена MCT8).
4. Синдромальные формы гипотиреоза.А. Синдром Пендреда (гипотиреоз, глухота, зоб) — мутация гена SLC26A4 (PDS) (дефект пендрина).Б. Синдром Бамфорда–Лазаруса (гипотиреоз, расщелина мягкого неба, волосы с острыми прядями) — мутация гена FOXE1 (TTF2).В. Кохера–Дебре–Семиланжа синдром (мышечная псевдогипертрофия, гипотиреоз).Г. Эктодермальная дисплазия, гипогидроз, гипотиреоз, цилиарная дискинезия.Д. Хореоатетозис, (гипотиреоз неонатальный, респираторный дистресс-синдром) — мутации генов NKX 2-1 (TTF1).Ж. Ожирение, колит, гипотиреоз — гипертрофия миокарда — задержка психического развития.
5. Транзиторный гипотиреоз.А. Прием матерью антитиреоидных препаратов.Б. Трансплацентарный перенос блокирующих антител к рецептору ТТГ.В. Дефицит или избыток йода у матери или новорожденного.Г. Гетерозиготные мутации генов DUOX2 или DUOXA2.Д. Врожденная гемангиома печени или гемангиоэндотелиома.

**Table:** Таблица 3. Дефекты генов, приводящие к врожденному гипотиреозу [1][3]Table 3. Gene defects leading to congenital hypothyroidism [1][3] АР — аутосомно-рецессивный тип наследования; АД — аутосомно-доминантный тип наследования; N — норма; ↑ — выше нормы; ↓ — ниже нормы.

	Частота встречаемости	Наследование	Ген	Зоб	T4	ТТГ	ТГ	Захват йода
Дисгенезия ЩЖ	1:4000	АР	NKX2-1, FOXE1, PAX-8	-	↓	↑	↓	↓
Семейный дефицит ТТГ	Редко	АР	TSHB, TSHA	-	↓	↓	↓	↓
Гипопитуитаризм	1:21 000	АР	PROP-1, Pit-1	-	↓	↓, N	↓	↓
Резистентность к ТТГ	Редко	АР	TSHR	-	↓	↑	↓	N
Дефект транспорта йода	Редко	АР	SLC5A5, SLC26A4	+	↓	↑	↑	↓
Дефект органификации йода	1:40 000	АР	ТPО	+	↓	↑	↑	N, ↑
Синдром Пендреда	1:50 000	АР	PDS	+	↑, N	↑	↑	N, ↑
Дефект синтеза ТГ	1:40 000	АР	ТG	+	↓	↑	↑, ↓	N, ↑
Дефект дейодиназы	Редко	АР	IYD	+	↓	↑	↑	N, ↑
Резистентность к ТГ	1:100 000	AP АД	THR-B	+	↑	↑, N	↑	↑
Резистентность к ТГ	Редко	АД	THR-A	-	N ↓, Т3 ↑	N	N, ↑	N

## ПРИЛОЖЕНИЕ Б.

## АЛГОРИТМЫ ДЕЙСТВИЙ ВРАЧА

**Figure:**
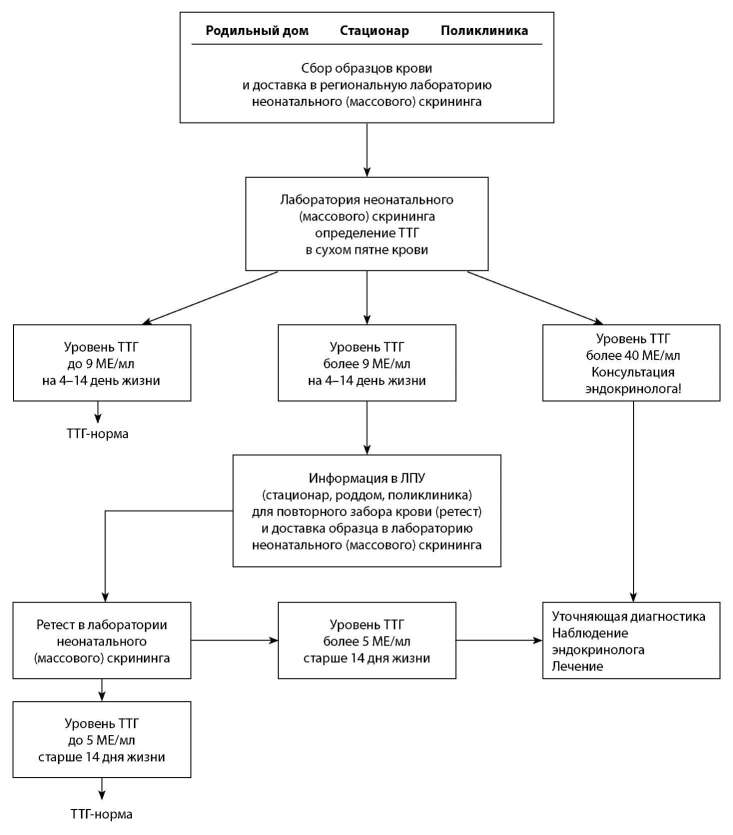
АЛГОРИТМ НЕОНАТАЛЬНОГО СКРИНИНГА НА ВРОЖДЕННЫЙ ГИПОТИРЕОЗ (ДОНОШЕННЫЕ ДЕТИ)

## ПРИЛОЖЕНИЕ Г1-Г2.

## ШКАЛЫ ОЦЕНКИ, ВОПРОСНИКИ И ДРУГИЕ ОЦЕНОЧНЫЕ ИНСТРУМЕНТЫ СОСТОЯНИЯ ПАЦИЕНТА, ПРИВЕДЕННЫЕ В КЛИНИЧЕСКИХ РЕКОМЕНДАЦИЯХ

ПРИЛОЖЕНИЕ Г1.ШКАЛА АПГАР ДЛЯ ДИАГНОСТИКИ ВРОЖДЕННОГО ГИПОТИРЕОЗА У НОВОРОЖДЕННЫХ

Название: Шкала Апгар для диагностики врожденного гипотиреоза у новорожденных.Источник: Дедов И.И., Петеркова В.А. Справочник детского эндокринолога. М.: Литтерра, 2020.. С. 98.Тип: шкала оценки.Назначение: оценка клинических симптомов для диагностики ВГ.

**Table:** Содержание (шаблон) и ключи интерпретации Пояснения: диагноз ВГ устанавливается при сумме баллов более 5.

Клинический признак	Количество баллов
Пупочная грыжа	2
Отечное лицо	2
Запоры	2
Женский пол	1
Бледность, гипотермия кожи	1
Увеличенный язык	1
Мышечная гипотония	1
Желтуха дольше 3 недель	1
Шелушение и сухость кожи	1
Открытый задний родничок	1
Беременность длилась более 40 недель	1
Масса тела при рождении более 3500 г	1

ПРИЛОЖЕНИЕ Г2.ТЕСТ ВЕКСЛЕРА (ДЕТСКИЙ ВАРИАНТ)

Название на русском языке: Тест Векслера (детский вариант).Оригинальное название Wechsler Intelligence Scale for Children, WISC.Источник (официальный сайт разработчиков): Тест Векслера: диагностика структуры интеллекта (детский вариант): методическое руководство/Ю.И. Филимоненко, В.И. Тимофеев. — Санкт-Петербург: ИМАТОН, 2016. — 106 с. — (ИМАТОН. Профессиональный психологический инструментарий) www.imaton.com.Тип: шкала оценки.Назначение: исследование структуры интеллекта у детей от 5 до 16 лет.

**Table:** Содержание (шаблон) и ключи интерпретация Пояснения: за каждый субтест пациент получает определенное количество баллов, в последующем бальная оценка переводится в шкальную в зависимости от возраста. В ходе тестирования определяется IQ общий — интегральный показатель, являющийся индикатором общего интеллекта, IQ вербальный — подструктура общего интеллекта, функционирование которой осуществляется в вербально-логической форме с преимущественной опорой на знания. IQ невербальный — также является подструктурой общего интеллекта, успешность выполнения данной части теста связана с умениями обследуемого, особенностями его психофизических, сенсомоторных и перцептивных характеристик.

WISC (для детей от 5 до 16 лет)
Вербальная часть	Невербальная часть
осведомленность	недостающие детали
понятливость	последовательность картинки
арифметический	кубики Коса
сходство	складывание фигур
словарный	шифровка
повторение цифр	лабиринты

Оценка интеллектуального развития (IQ)	Уровень
менее 70	умственная отсталость
70-79	пограничный уровень
80-89	сниженная норма интеллекта
более 90	норма
90-109	средний уровень
110-119	хорошая норма
120-129	высокий интеллект
более 130	весьма высокий
